# The effect of livestock grazing on plant diversity and productivity of mountainous grasslands in South America – *A meta‐analysis*

**DOI:** 10.1002/ece3.11076

**Published:** 2024-04-15

**Authors:** Ana Patricia Sandoval‐Calderon, Nerea Rubio Echazarra, Marijke van Kuijk, Pita A. Verweij, Merel Soons, Yann Hautier

**Affiliations:** ^1^ Ecology & Biodiversity Group, Department of Biology Utrecht University Utrecht The Netherlands; ^2^ Herbario Nacional de Bolivia (LPB) San Andres University La Paz Bolivia; ^3^ Copernicus Institute of Sustainable Development Utrecht University Utrecht The Netherlands

**Keywords:** grasslands, grazing exclusion, livestock, meta‐analysis, mountains, plant diversity, productivity, South America

## Abstract

Mountainous grasslands in South America, characterized by their high diversity, provide a wide range of contributions to people, including water regulation, soil erosion prevention, livestock feed provision, and preservation of cultural heritage. Prior research has highlighted the significant role of grazing in shaping the diversity and productivity of grassland ecosystems, especially in highly productive, eutrophic systems. In such environments, grazing has been demonstrated to restore grassland plant diversity by reducing primary productivity. However, it remains unclear whether these findings are applicable to South American mountainous grasslands, where plants are adapted to different environmental conditions. To address this uncertainty, we conducted a meta‐analysis of experiments excluding livestock grazing to assess its impact on plant diversity and productivity across mountainous grasslands in South America. In alignment with studies in temperate grasslands, our findings indicated that herbivore exclusion resulted in increased aboveground biomass but reduced species richness and Shannon diversity. The effects of grazing exclusion became more pronounced with longer durations of exclusion; nevertheless, they remained resilient to various climatic conditions, including mean annual precipitation and mean annual temperature, as well as the evolutionary history of grazing. In contrast to results observed in temperate grasslands, the reduction in species richness due to herbivore exclusion was not associated with increased aboveground biomass. This suggests that the processes governing (sub)tropical grassland plant diversity may differ from those in temperate grasslands. Consequently, further research is necessary to better understand the specific factors influencing plant diversity and productivity in South American montane grasslands and to elucidate the ecological implications of herbivore exclusion in these unique ecosystems.

## Graphical abstract

Mountainous grasslands in South America hold high diversity, provide a wide range of contributions to people, including water regulation, soil erosion prevention, livestock feed provision, and preservation of cultural heritage. Processes governing (sub)tropical grassland plant diversity may differ from those in temperate grasslands. Further research is necessary to better understand the specific factors influencing plant diversity and productivity in South American montane grasslands.

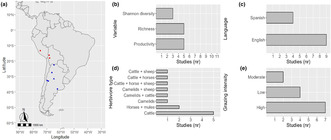

## 1 INTRODUCTION

Natural and semi‐natural grasslands cover 40% of Earth's land surface and provide habitat and food for billions of animals and humans (Lemaire et al., 2011; Suttie et al., 2005). Mountainous grasslands comprise around 2% of all the grasslands in the world (Arasumani et al., 2021). Despite their relatively small size and low productivity, mountainous grasslands exhibit high biodiversity and offer a wide range of contributions to people. In South America, mountainous grasslands account for approximately 10% of the total sub‐continent land cover (Eva et al., 2004). These grasslands play a vital role in providing water, food, fuel, and genetic resources (Christmann & Menor, 2021). Additionally, they contribute significantly to climate regulation by sequestering carbon in the soil (Boval et al., 2017). Despite their invaluable functions, these grasslands stand out globally for exhibiting the highest erosion values, with 53.2% of them experiencing elevated erosion rates (10 Mg/ha/year) (Straffelini et al., 2024). Nevertheless, the impact of livestock grazing on the biodiversity and functioning of South American mountainous grasslands remains uncertain. Unraveling this impact is crucial for ensuring the long‐term conservation of these ecosystems and sustaining their contributions to local communities.

Studies on the impact of grazing usually involve grazing exclusion experiments comparing (naturally) grazed to (experimentally) ungrazed sites. Multiple global meta‐analysis and collaborative experimental studies assessing the impact of grazing exclusion on plant diversity and productivity have yielded contrasting results. In most studies, grazed plots usually have higher diversity, when compared to exclusions (Borer et al., 2014; Gao & Carmel, 2020; Lezama et al., 2014), but some studies report small effects on average (Herrero‐Jáuregui & Oesterheld, 2017). The positive response of biodiversity to grazing is usually attributed with ground‐level light availability as the common process modulating the relationships among plant diversity, herbivory, and plant productivity (Borer et al., 2014, 2017; Eskelinen et al., 2022). Indeed, previous studies have shown that herbivores can maintain diversity in nutrient‐rich grasslands by removing biomass and alleviating light‐limitation in the lower vegetation layers, particularly in more humid, productive ecosystems (Bakker et al., 2006; Borer et al., 2014). However, alleviating light limitation through herbivory may not be the only process changing plant diversity (Eskelinen et al., 2022). Several other factors not depending on biomass removal, such as seed dispersal, trampling, and destruction of root systems, may also contribute to changes in diversity due to herbivory (Eskelinen et al., 2022). For example, livestock trampling can physically fragment plant and litter material on the ground and reduce surface coverage which may affect plant diversity (Wei et al., 2021). In addition, herbivores can also be vectors for plant dispersal via consumption and egestion of seeds or attachment of seeds to fur (Cosyns et al., 2005; Malo & Suárez, 1995).

Additionally, the impact of herbivory on plant diversity and productivity may be also modulated by grazing intensity (Zhou et al., 2017), the duration of grazing exclusion (McSherry & Ritchie, 2013), the evolutionary history of grazing (Milchunas et al., 1988), and climatic conditions (Bai et al., 2012). First, moderate grazing levels can have a positive impact on diversity, while no grazing or heavy grazing may decrease diversity (i.e., intermediate disturbance hypothesis, Connell, 1978; Milchunas et al., 1988). Second, short‐term exclusion of grazers (4–5 years) can significantly increase biodiversity (Hu et al., 2016), but long‐term exclusion (>10 years) may decrease plant diversity (Song et al., 2020). Third, grasslands with long evolutionary history of ungulate grazing (>500–10,000 years) usually show a negative relationship between species richness and herbivore exclusion, while no relationship is found in grasslands with short evolutionary history of grazing (<500 years) (Cingolani et al., 2005; Milchunas et al., 1988; Price et al., 2022). Finally, regional climatic conditions can result in varying responses to grazing (Maestre et al., 2022). For example, in grasslands with high precipitation, grazing exclusion generally decreases plant diversity (Price et al., 2022). In contrast, in arid regions, grazing exclusion tends to play a positive role in maintaining species richness (Gao & Carmel, 2020). The positive impact on diversity is usually related to increases in temperature and precipitation during the peak growing season (Su et al., 2023). Importantly, most studies, meta‐analyses, and coordinated experiments on grazing exclusion have focused on temperate grasslands of North America, Europe, or Asia. Consequently, grasslands in the tropics and subtropics are understudied (Christmann & Menor, 2021), and to our knowledge, no prior synthesis efforts have been made in the (sub)tropical regions of South America. In (sub)tropical mountainous areas, a consistently mild to warm climate prevails throughout the year, fostering the growth of diverse vegetation. Subtropical mountainous regions can exhibit cooler conditions compared to their tropical counterparts (Kohler et al., 2014). However, like tropical zones, subtropical mountainous areas encompass a variety of elevations, leading to diverse vegetation zones and ecosystems. The transition between tropical and subtropical zones in mountainous regions may lack clear boundaries, resulting in ecological overlap and complexity (Martin et al., 2011). Whether the knowledge derived from studies conducted in temperate grasslands applies to other types of grasslands remains unclear. Temperate and (sub)tropical mountainous grasslands are characterized by different pools and abundances of plant species. Temperate grasslands typically contain both short‐statured species that are tolerant of grazing and tall‐stature species that are susceptible to grazing but excel in light capture (Milchunas et al., 1988). The dominance of these species varies along a productivity gradient, which is determined by changes in limiting resources from low‐ to high‐productivity areas (Newman, 1973; Tilman, 1982). In contrast, (sub)tropical mountainous grasslands in South America predominantly consist of short stature species with exceptionally high ratio of above to belowground biomass (Patty et al., 2010; Smith & Klinger, 1985). This high ratio might result in extensive coverage of short stature vegetation, limited recovery after herbivore exclusion, increased potential for degradation, and reduced productivity, particularly in areas with a long history of grazing (Sarmiento, 1986). For example, observational studies have reported a significant degradation of South American mountainous grasslands with losses of biodiversity and productivity in 60%–80% of its area, primarily due to overgrazing (Hofstede, 1995; Molinillo & Monasterios, 1997; Suttie et al., 2005; Verweij, 1995).

Here, by conducting a comprehensive meta‐analysis of grazing exclusion studies in mountainous grasslands of South America, we aim to provide a deeper understanding of the causal impact of livestock grazing on plant diversity and productivity and contribute to the formulation of more effective environmental policies in the region. Specifically, we investigate the effect of grazing exclusion on biomass, species richness, and Shannon diversity index. Next, we address the influence of duration of exclusion on aboveground biomass and in plant diversity. Finally, we analyze the effect of grazing exclusion on plant diversity under different grazing intensities and with distinctive climatic conditions and history of grazing. We expect that (i) grazing exclusion increases biomass but reduces species richness and Shannon diversity index, thus leading to a negative relationship between biomass and diversity; (ii) higher duration of exclusion increases aboveground biomass and in turn decreases plant diversity; and (iii) grazing exclusion increases plant diversity only under moderate grazing intensities and/or in sites with drier conditions and with a shorter history of grazing.

## 2 MATERIALS AND METHODS

### 2.1 Literature search

**FIGURE 1:**
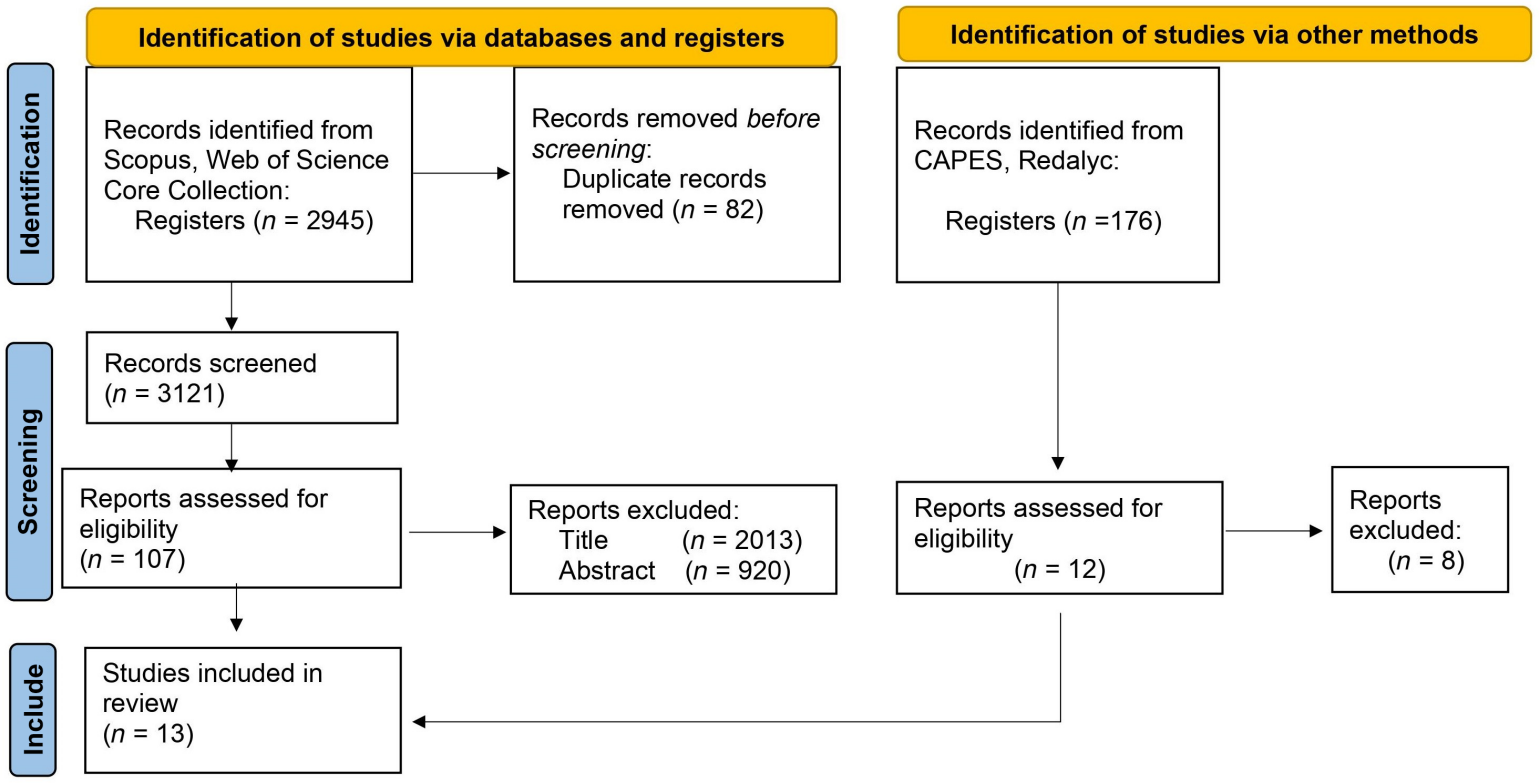
Prisma statement; flow of information through the different phases of the systematic literature search.

We conducted a meta‐analysis of specific relationships following the Preferred Reporting Items for Systematic Reviews and Meta‐Analyses (PRISMA) (Moher et al., 2009) as much as possible. First, we used the PICO framework (Foo et al., 2021) to identify relevant literature and to obtain a search string as inclusive as possible. This approach identifies “PICO elements” in a research question: population or subject, intervention, comparator, and outcomes. Appendix S1 shows the PICO elements for the definition of our search string. Then, international academic literature databases Scopus, Web of Science Core Collection, and Scientific Electronic Library Online (SciELO) were used to assess the literature. In particular, we alleviate previous limitations by including publications in South American scientific journals: CAPES and Redalyc. Lastly, we conducted a Reference and Citation search. For the South American journals, we selected only categories related to biology, ecology, environmental sciences, and agricultural sciences. These journals accepted the designed search string containing the Booleans AND and OR. However, when the results were retrieved, it was noticed that the filters were not effective and publications from other disciplines were obtained alongside the indicated disciplines. Therefore, in CAPES, the first 500 results were screened. In the case of Redalyc, the search was conducted per country (i.e., Perú, Bolivia, Colombia, Venezuela, Chile, Argentina, and Ecuador); from each of them, the first 500 results were screened. The search was conducted in Spanish and English. The search string used for both languages had the same main structure, using Boolean operators and key terms. We identified 3121 potential publications to start the screening process (search strings are provided in Appendix S2). Initially, the titles and/or abstracts of these publications were scanned; this resulted in 2937 records excluded. After the initial scan indicated that the publication was potentially relevant (i.e., 102 records), the full text was scanned. In total, 13 studies were found that met the inclusion criteria of the literature search. This process is gathered in the Prisma statement (Figure 1).

During the screening process, we defined the following inclusion criteria: (i) Relevant subject(s): Natural and semi‐natural grasslands in tropical and subtropical mountainous areas of South America. Grasslands are defined here as areas with little or no trees and a high proportion of graminoid and forb species (>50% graminoid and herbaceous cover before treatments). Studies conducted in mountainous grasslands located within the subtropical latitudes (between 23.45° and approximately 36° in the Northern and Southern hemispheres) were also included if they had the following climate characteristics: (1) low average temperatures but with marked diurnal variations, bigger than the annual temperature variation, (2) rainfall seasonality with a dry and a wet season, and (3) high levels of solar radiation. (ii) Types of intervention: Plots that did not receive grazing; that is, exclusion of herbivores by fences. (iii) Types of comparators: Grazing by mammalian herbivore ungulates (alone or in species combination). (iv) Types of outcomes: Plant species richness, Shannon diversity, and/or aboveground biomass. (v) Types of study: Scientific journal articles and book chapters, only peer‐reviewed experimental studies.

It is important to note that the database initially included 15 studies; however, during the analysis process, we removed two of them. First, two studies were found to be almost identical: Pucheta, Cabido, et al. (1998) and Pucheta, Vendramini, et al. (1998) are based on the same data collection but published in different languages – English and Spanish. We kept Pucheta, Cabido, et al. (1998) because it reported a higher sample size, and the data were easier to extract. Second, we removed Oliveras et al. (2014), because productivity was reported as Mg C ha^−1^ year^−1^ and the information given was not detailed enough to compare it with our unit of biomass (g ha^−1^). Most of the final number of publications included in the meta‐analysis reported more than one grazing versus exclusion comparison (hereafter comparison or paired comparison), because: (i) they had more than one grazing‐exclusion site or (ii) they studied the effect of grazing exclusion in different years (increasing exclusion duration) and at different grazing intensities. This resulted in 13 studies (Appendix S3) and 27 paired comparisons (Appendix S4); however, not all of them reported the three response variables of interest (i.e., Shannon diversity, species richness, and aboveground biomass).

### 2.2 Data extraction

All included publications reported a mean measure of species richness and/or Shannon diversity and/or aboveground biomass and a corresponding standard deviation in both exclusion and grazing conditions. In case a standard deviation estimate was not given (24 comparisons), we calculated it using the standard error estimate and sample size (17 comparisons). If no standard error estimate was given, we took 1/10 of the mean measure (7 comparisons). When the study did not explicitly report results but instead presented data in a figure, we extracted the mean and corresponding error of the response variable using GetData Graph Digitizer (2.26 version, Fedorov, 2013). We included data from all grazing sites (or each paired comparison) if studies had more than one grazing site (or more than one paired comparison).

We extracted the following explanatory variables from the selected studies: publication year, country, study site, latitude, longitude, language of the publication, climatic zone, exclusion duration (in years), herbivore type, grassland type, evolutionary history of grazing, and grazing intensity. We contacted authors via email, if necessary, when data were lacking from the publication. We were unable to use quantitative grazing intensity (i.e., livestock load), because none of the selected studies reported the carrying capacity of the study site. Animal unit per hectare (AU/ha), without the carrying capacity, would not have been comparable during the meta‐analysis. Therefore, grazing intensity was taken as a categorical variable with three categories: low, moderate, and high grazing intensity based on the qualification by the authors of the study. If the study reported extensive grazing or overgrazing, it was considered as low and high grazing intensity, respectively. The studies by Pucheta, Cabido, et al. (1998) and Pucheta et al. (1992) did not report the intensity of the livestock load. However, their study site was the same as Marquez et al. (2002) and Nai‐Bregaglio et al. (2002), and therefore, the livestock loads were compared, and grazing intensity was deduced. The evolutionary history of grazing was treated as a categorical variable, classified into two categories: short (less than 500 years of grazing) and long grazing history (exceeding 500 years of grazing). Notably, domestic camelids, such as llamas or alpacas, have a well‐documented history dating back more than 5000 years in the Central and Southern Andean mountains. However, the introduction of European livestock has led to the disappearance of camelid grazing from Southern areas, with their presence now confined to the tropical zone of the Andes (Vilá & Arzamendia, 2022). In our categorization, we considered grazing history as long at study sites within the tropical climatic zone dominated by camelids. Conversely, we considered grazing history as short at study sites within the subtropical climatic zone dominated by cattle, sheep, or horses. This differentiation is crucial because camelids exhibit less trampling compared to the other grazers, resulting in reduced soil compaction (Zimmer et al., 2023). For the grassland type, some conversion had to be made: (i) from “Pampa,” “Jarillal,” “Mountain grassland,” and “Pastizal en filo” to tall grassland and (ii) from meadow and peatland to bofedal.

### 2.3 Meta‐analysis response ratio and statistics

The effect sizes of grazing exclusion on species richness, Shannon diversity index, and aboveground biomass were calculated between the 27 paired comparisons using the log response ratio (LRR) method. We decided to use LRR because it is a statistical tool that enhances the comparability, stability, and interpretability of data when assessing the effects of experimental treatments or conditions (Bakbergenuly et al., 2020; Borenstein et al., 2009). For each reported comparison, the LRR was calculated following the same methods as Hedges et al. (1999) and Luo et al. (2006). The LRR quantifies the proportional change after an experimental manipulation (Hedges et al., 1999), which in this case was livestock exclusion. We calculated the LRR as:

$$\mathrm{LRR}=\ln\left(\mathrm{Xt}/\mathrm{Xc}\right).$$

in which *Xt* and *Xc* are the mean values of the exclusion treatment and control group (grazing), respectively. The natural logarithm of the LRR is used because if *Xt* and *Xc* are normally distributed and both are bigger than zero, then $\ln\left(\mathrm{Xt}/\mathrm{Xc}\right)$ will approximately be normally distributed (Luo et al., 2006). The variance (*v*) of the LRR is calculated as:

$$v=\frac{s_{t}^{2}}{n_{t}{\bar{x}}_{t}^{2}}+\frac{s_{c}^{2}}{n_{c}{\bar{x}}_{c}^{2}}$$

in which 𝑛_𝑡_ and 𝑛_𝑐_ are the sample sizes, and 𝑠_𝑡_ and 𝑠_𝑐_ the standard deviations of the exclusion treatment and the control group (grazing treatment), respectively. As in Luo et al. (2006), the weighted response ratio (RR++) from individual RR_*𝑖j*_ (𝑖 = 1, 2, …, 𝑚; 𝑗 = 1, 2, …, 𝑘) was calculated by giving more weight to those studies with higher precision estimates, lower variance (𝑣), which resulted of a more precise combined estimate and a greater power of the tests. 𝑚 is the total number of groups (e.g., climatic zones, grazing history, different grazing intensities), and 𝑘 is the number of paired comparisons. The formulas to calculate the weighted mean response ratio (RR++) and the weighted standard error (S(RR++)) are:

$${\mathrm{RR}}_{++}=\frac{\sum_{i=1}^{m}\sum_{j=1}^{k}w_{\mathrm{ij}}{\mathrm{RR}}_{\mathrm{ij}}}{\sum_{i=1}^{m}\sum_{j=1}^{k}w_{\mathrm{ij}}}\;S\left({\mathrm{RR}}_{++}\right)=\sqrt{\frac{1}{\sum_{i=1}^{m}\sum_{j=1}^{k}w_{\mathrm{ij}}}}$$

in which *w*_*ij*_ is the weighting factor and is calculated as:

$${\text{34𝑤}}_{\text{34𝑖j}}=\text{1/34𝑣.}$$

The 95% confidence interval (CI) for the LRR is:

$$95\%\mathrm{CI}=\mathrm{RR}++\pm 1.96\;\left(\mathrm{RR}++\right).$$

The effect of grazing was considered as significant if the *p*‐value was smaller than .05 and if the 95% CI of RR++ did not overlap with zero. Significant results were reported as a percentage of change calculated as:

$$\%\text{change}={\text{(34𝑒}}^{\text{34𝑒𝑅34𝑒𝑅𝑅++}}-1)\times 100\%.$$

The between‐study heterogeneity was assessed and reported using Higgins & Thompson's *I* ^2^ statistic that describes the percentage of variation across studies that is due to heterogeneity rather than chance (Higgins & Thompson, 2002; Higgins et al., 2003).

### 2.4 Meta‐regression and subgroup analysis

We performed meta‐regression analysis using exclusion duration as the predictor. The relationship between LRR of total aboveground biomass and LRR of species richness was also calculated. The relationship between LRR of total aboveground biomass and LRR of Shannon diversity could not be assessed because the sample size was too small (*n* = 4, case studies number = 16). All statistical analyses were conducted in R, using the packages “meta.” Effect sizes (LRR) per response variable were visualized using forest plots, meta‐regression for exclusion duration was visualized using bubble plots, and for the remaining plots the package “ggplot2” was used. Lastly, sub‐group analysis was conducted to determine the extent of the difference between the subgroups and to test if certain explanatory variables have an influence on the effect of exclusion or not. More specifically, subgroup analyses were conducted with two explanatory variables: climatic zone (tropical and subtropical) and grazing intensity (high, moderate, and low).

## 3 RESULTS

### 3.1 Descriptive analysis of the studies found in the literature

**FIGURE 2:**
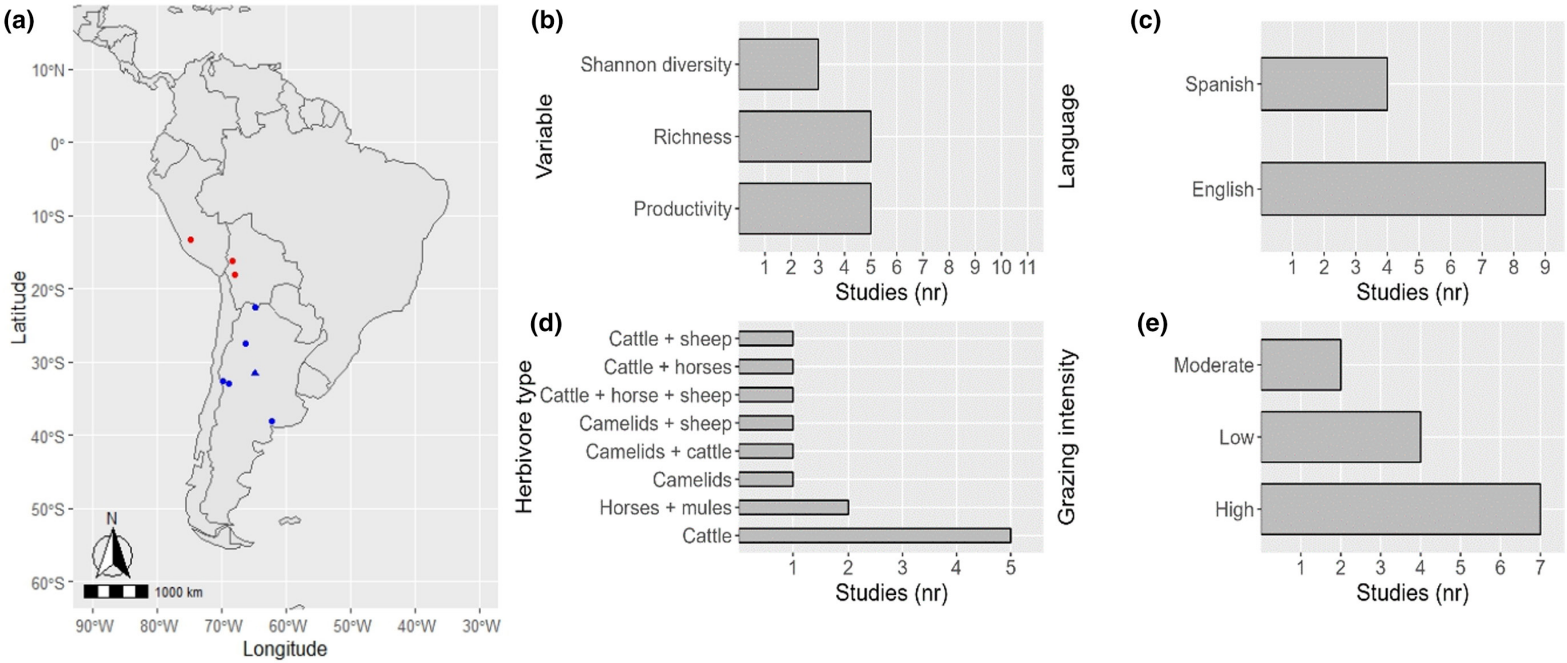
Overview of experimental studies on the effect of herbivory exclusion on biodiversity in mountainous grasslands communities. (a) Map of South America indicating the location of each study site. The locations within the tropical region are marked in red, while those within the subtropical region are marked in blue. The circles are locations with one study and the triangle a location with five studies. (b) Frequency of the reported response variables. (c) Frequency of the language used in the publications. (d) Frequency of the reported herbivore type. (e) Frequency of the grazing intensities. Appendix S4 provides a more comprehensive overview of variables considered for each data point.

We found 13 studies that matched our search criteria. Ten studies were conducted in Argentina, two in Bolivia and one in Perú. Figure 2a shows the location of each study. The response variables that were reported the most were plant species richness (*n* = 5) and productivity (*n* = 5), followed by Shannon diversity index (*n* = 3) (Figure 2b). Four studies were in Spanish and nine in English (Figure 2c). Most of the studies were conducted in grasslands with only cattle grazing (*n* = 5), one study had camelid grazing, and the remaining had mixed herds (Figure 2d). Seven studies had high grazing intensity, two had moderate intensity, and four had low intensity (Figure 2e). Lastly, exclusion duration ranged from 0 (initial state) to 15 years, with a median of 2.

### 3.2 Effect of livestock exclusion on species richness, Shannon diversity, and total aboveground biomass

**FIGURE 3:**
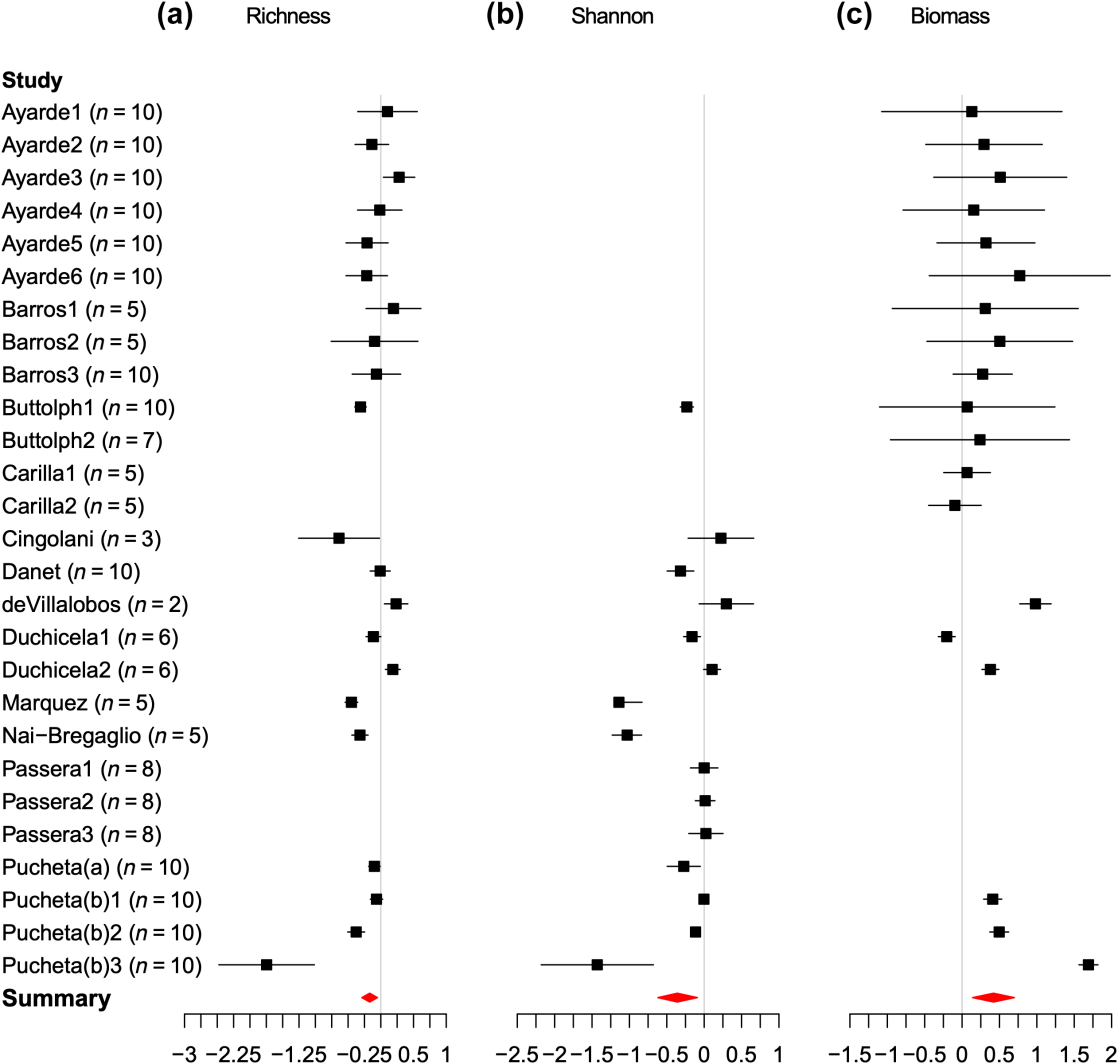
Individual and summary effect sizes of grazing exclusion of the 27 paired comparisons, calculated as the natural logarithm of the ratio (lrr) of the variable within the grazing exclusion plot divided by the average of the variable in the reference plots (±95% confidence intervals) of the studies reporting (a) species richness (*I* ^2^ = 84%, *p* < .0001), (b) Shannon diversity index (*I* ^2^ = 94%, *p* < .0001), and (c) aboveground biomass (*I* ^2^ = 96%, *p* < .0001).

The meta‐analysis showed that overall, herbivore exclusion decreased species richness (mean and 95% CIs = −0.14 (−0.25, −0.02)) (Figure 3a) and Shannon diversity (mean and 95% CIs= = −0.24 (−0.46, −0.01)) (Figure 3b) conversely increased aboveground biomass (mean and 95% CIs = 0.41 (0.16, 0.66) (Figure 3c)).

### 3.3 Relationship between plant species richness and aboveground biomass under grazing exclusion

**FIGURE 4:**
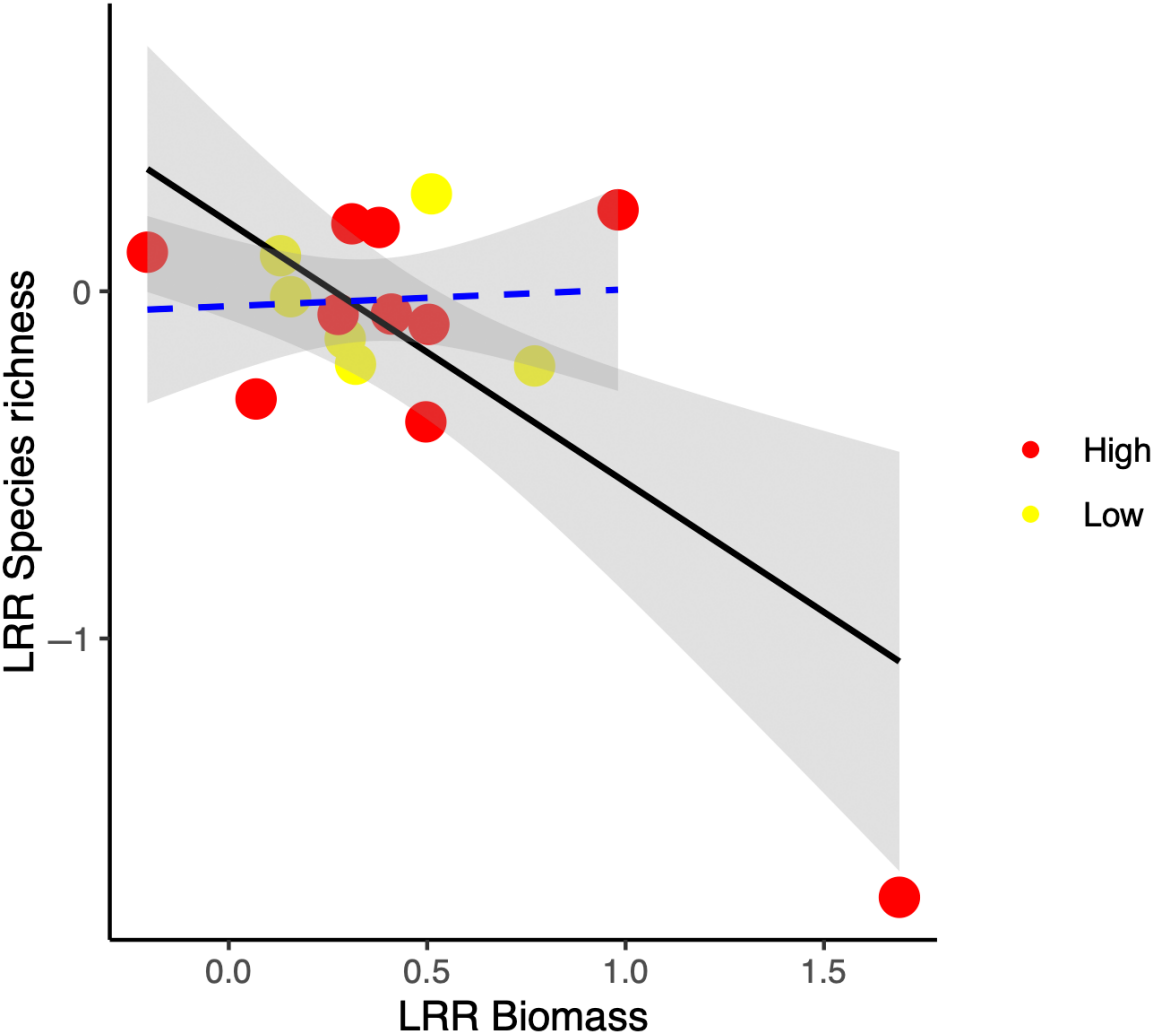
Effect of herbivore exclusion on species richness as mediated by changes in aboveground biomass. The black line represents the linear regression including all data points and the blue line represents the linear regression excluding the study with the longest duration. The solid line represents a significant relationship and the dashed line a non‐significant relationship.

We found that changes in species richness in response to exclusion of herbivores was negatively related to changes in aboveground biomass (slope and 95% CIs = −0.75 (−1.21, −0.30)) (black line in Figure 4). However, this negative relationship was mostly due to one study with the longest duration of exclusion (15 years). When this single study was removed from the analysis, we found no relationship between changes in aboveground biomass and changes in species richness (slope and 95% CIs = 0.05 (−0.38, 0.48), blue line in Figure 4).

### 3.4 Meta‐regression: Influence of exclusion duration on the effect of livestock exclusion on Shannon diversity, species richness, and total aboveground biomass

**FIGURE 5:**
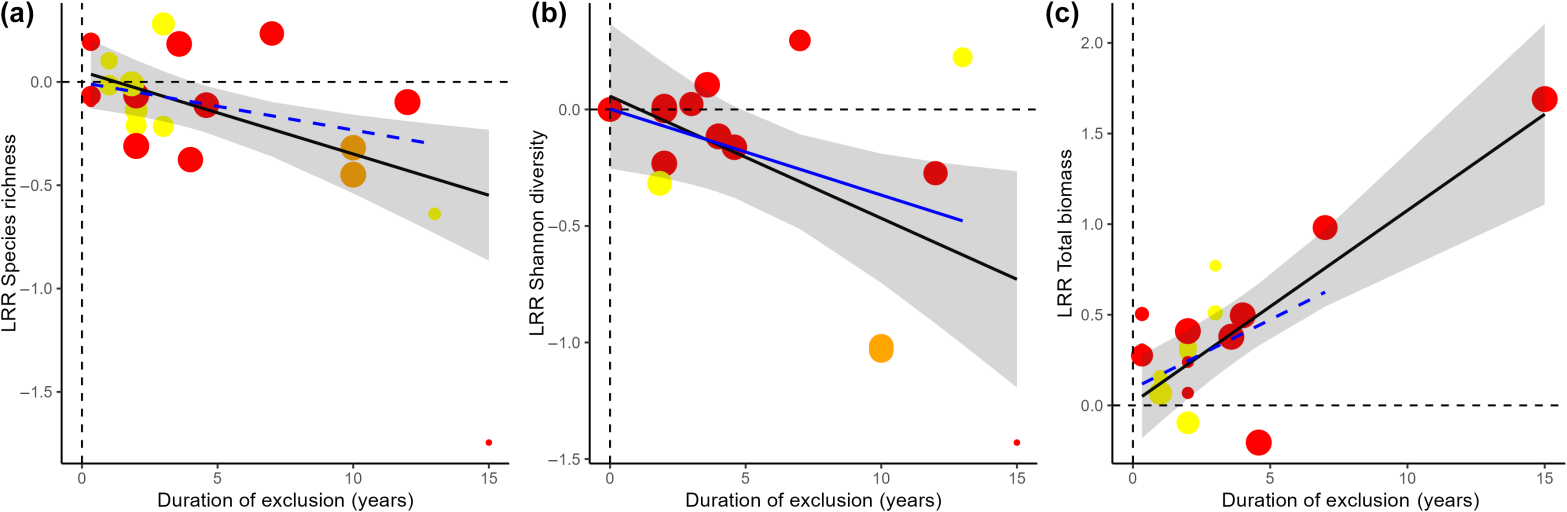
Individual effect sizes of grazing exclusion, calculated as the natural logarithm of the ratio (lrr) of the variable within the treatment plot divided by the average of the variable in the reference plots (±95% confidence intervals) against exclusion duration for (a) plant species richness, (b) Shannon diversity index, and (c) total aboveground biomass. The black lines represent the linear regression including all data points and the blue lines represent the linear regression excluding the study with the longest duration. Solid lines represent significant relationships and dashed lines non‐significant relationships. The size of the points is proportional to the weight that the reported comparisons have received in the analysis.

Based on the effect sizes of the paired comparisons, the meta‐regression showed that increasing exclusion duration led to a decrease in both species richness (slope and 95% CIs = −0.04 (−0.07, −0.01)) and Shannon diversity (slope and 95% CIs = −0.05 (−0.10, −0.01)), but an increase in aboveground biomass (slope and 95% CIs = 0.11 (0.06, 0.15)) (black lines in Figure 5a‐c). The negative impacts of increasing exclusion duration on species richness and aboveground biomass were mostly due to the inclusion of the study with the longest duration, as they did not remain significant when this data point was removed from the analysis (blue dashed lines in Figure 5a slope and 95% CIs = −0.02 (−0.05, 0.01) and 5c slope and 95% CIs = 0.08 (−0.02, 0.17)).

### 3.5 Subgroup analysis: Influence of climatic zone, evolutionary history of grazing, and grazing intensity on the effect of livestock exclusion on species richness, Shannon diversity, and aboveground biomass

The effects of livestock exclusion on species richness, Shannon diversity, and aboveground biomass did not depend on climatic zone (subtropical and tropical zone, Table 1), evolutionary history of grazing, and grazer type (Appendix S4). In contrast, we found that the effects of livestock exclusion on species richness, Shannon diversity, and aboveground biomass depend on grazing intensity. Specifically, livestock exclusion decreased species richness and Shannon diversity under moderate grazing (*n* = 2) and above‐ground biomass increased under high grazing intensity.

**TABLE 1:** Estimated effect and heterogeneity in each subgroup, as well as the *p‐*value of the test for subgroup differences for Shannon diversity, species richness, and aboveground biomass.

Response variable	Subgroups	*n*	Effect size	95% CI	*p*_subgroup_
Species richness	Climatic zone				
	Subtropical	11	−0.16	−0.31, −0.01	.46
	Tropical	4	−0.06	−0.27, 0.14	
	Grazing intensity				
	High	11	−0.13	−0.34, 0.07	<.0001
	Moderate	2	−0.39	−0.52, −0.26	
	Low	8	−0.05	−0.20, 0.09	
Shannon diversity	Climatic zone				
	Subtropical	11	−0.27	−0.59, 0.04	.48
	Tropical	4	−0.15	−0.32, 0.03	
	Grazing intensity				
	High	11	−0.07	−0.16, 0.02	<.0001
	Moderate	2	−1.03	−1.16, −0.89	
	Low	2	−0.32	−0.61, 0.43	
Biomass	Climatic zone				
	Subtropical	15	0.50	0.21,0.78	.12
	Tropical	4	0.10	−0.31,0.51	
	Grazing intensity				
	High	11	0.52	0.16, 0.87	.04
	Low	8	0.10	−0.10, 0.29	

## 4 DISCUSSION

Our study is the first attempt to summarize the effects of grazing exclusion on Andean grasslands' plant diversity and productivity. Our results show an overall reduction in species richness and Shannon diversity index and increased aboveground biomass with livestock exclusion in mountainous grasslands in South America. Moreover, we found that the effects were stronger under longer duration of exclusion. However, we did not find a link between the change in species richness and aboveground biomass in response to grazing, and a weak relationship of grazing intensity with diversity and aboveground biomass.

### 4.1 Descriptive analysis of the studies found in the literature

Our analyses are based on a total of 13 studies, of which 4 are studies published in Spanish. The low number of studies found underscores the scarcity of research conducted in (sub‐)tropical mountainous grasslands and stresses the critical need to include studies reported in the Spanish language into our understanding of these ecosystems. Data on tropical mountainous grasslands are rare, these ecosystems span across all continents in the tropical belt, and despite their small spatial extent of just below 1 million km^2^ which accounts for less than 1% of the global grassland cover, they contribute to millions of people worldwide (Christmann & Menor, 2021).

### 4.2 Effect of livestock exclusion on Shannon diversity, species richness, and total aboveground biomass

We found that grazing exclusion decreased species richness and Shannon diversity index but increased aboveground biomass. These results are in line with previous studies. On a global scale, Gao and Carmel (2020) showed that grazing exclusion significantly decreased species richness. Similarly, Lezama et al. (2014) found that herbivore exclusion significantly decreased plant diversity, particularly in more humid, productive ecosystems in grasslands within a productivity gradient in South America. Milchunas and Lauenroth (1993), in 152 ungrazed–grazed contrasts around the world found that herbivore exclusion acts mainly on species composition through a turnover of species with a much smaller net change in species richness and diversity indices. Similarly, here we found a higher percentage of change in Shannon diversity compared to species richness. Finally, increases in aboveground biomass under herbivore exclusion are substantially supported by other meta‐analyses (Hao & He, 2019; Li et al., 2021; Liu et al., 2020).

### 4.3 Relationship between aboveground biomass and species richness under grazing exclusion

We did not find a link between the increase in biomass and decreases in plant diversity in response to removal of herbivores. This result contrasts with previous findings (Borer et al., 2014; Eskelinen et al., 2022) reporting that increased biomass under herbivore exclusion leads to reduced plant diversity. This loss of diversity is usually attributed to increased light competition following increased aboveground productivity. Eskelinen et al. (2022) experimentally demonstrated that the loss of plant diversity caused by livestock exclusion was mitigated by the addition of light. Our results suggest that other biomass‐independent processes could be more relevant in the sites studied in our meta‐analysis. We see several potential explanations that can explain this difference in results between ours and previous studies. First, both Borer et al. (2014) and Eskelinen et al. (2022) studied highly productive, eutrophic systems in temperate regions. The species composition in temperate grasslands varies in dominance along a productivity gradient and is determined by changes in limiting resources from low‐ to high‐productivity areas (Newman, 1973; Tilman, 1982). Thus, herbivores can act to maintain local‐scale plant diversity if they selectively consume the superior resource competitors (Borer et al., 2014). In contrast, the species composition in the studied (sub)tropical mountainous grasslands in South America consist mainly of short stature species and are less eutrophicated with lower productivity. Under such conditions, competition for light might not be the primary factor driving plant diversity (Hautier et al., 2018), instead reduced plant richness is likely to be driven by herbivores preferentially selecting rare palatable species (Lezama et al., 2014).

Second, livestock trampling could explain the changes in diversity by physically fragmenting plant and litter materials on the ground and reducing ground coverage (Wei et al., 2023). This means that grazing regulation on biodiversity at the study sites might depend strongly on soil resource conditions as seen in other studies (Eldridge et al., 2017; Li et al., 2018; Zhou et al., 2017). Under heavy grazing, trampling causes higher soil compaction, and lower soil porosity and water content, which accelerates soil surface evaporation and wind erosion, and reduces soil organic carbon levels (Rietkerk et al., 2000). This can negatively affect the abundance of vegetation. However, moderate trampling might help the organic matter to bind to the soil and reduce the negative effect on plant diversity; therefore, trampling as an intermediate frequency disturbance could promote competitive exclusion and colonization by less competitive species (Hobbs & Huenneke, 1992).

Third, vegetation at high elevations is prone to colonization from the local species pool and even high species richness may not constrain ingression of new species. Consequently, natural grazing by mammal herbivores favors species colonization and seedling emergence (Eskelinen & Virtanen, 2005). These characteristics are in line with our results, suggesting that mountainous grasslands could be dispersal‐assembled, meaning that most species are not stably coexisting but, instead, are transiently co‐occurring and are reliant on continued immigration (Loke & Chisholm, 2023). Finally, we could only include studies that had data on both species richness and aboveground biomass data, leaving us with a subset of the pair comparison data (*n* = 8). This limited the power of the analyses. Nevertheless, all the mentioned processes which are not linked to biomass removal by herbivores need to be better explored and need more attention, especially in grasslands with different characteristics as the mountainous grasslands in (sub) tropical regions.

### 4.4 Influence of exclusion duration on the effect of livestock exclusion on Shannon diversity, species richness, and total aboveground biomass

The meta‐regressions showed that the duration of the exclusion had a negative relationship with plant diversity and positive relationship with above‐ground biomass. However, once the influential value was removed from the analysis, only Shannon diversity showed a significant relationship with duration of exclusion. The influential value corresponds to 15 years of exclusion of the study by Pucheta, Cabido, et al. (1998), which was the only long‐term study where species richness and total aboveground biomass was reported. These results are in line with previous meta‐analyses showing small changes in species richness in response to disturbances compared to species composition (Herrero‐Jáuregui & Oesterheld, 2017). This suggests that changes in community composition are rapidly taking place while gain or extinction of species takes a longer duration to manifest (Milchunas & Lauenroth, 1993). This difference is most likely due to changes in abundance of species with particular traits in grazing treatments (Duchicela et al., 2020). For example, previous studies in South American mountainous grasslands have shown that grazing is associated with a reduction in dominance of palatable species, such as short grasses species with higher specific leaf area (SLA) and an increase in abundance of tolerant species, with higher leaf dry matter content (LDMC) such as cushion and prostate grasses (Diaz et al., 2004; Sandoval‐Calderon et al., 2023).

Similar to species richness, after removing the only long‐term study in our data set, we did not find a relationship between the duration of exclusion and the effect sizes of aboveground biomass. This result suggests that longer‐term studies are needed to determine the impact of herbivore exclusion on aboveground biomass. This is in line with studies in Chinese mountainous grasslands, where longer exclusion periods (exceeding 10 years) were needed to observe significant changes in aboveground biomass (Du et al., 2020; Jing et al., 2019). It is well‐established that herbivore exclusion can modify the abiotic environment inside exclusions compared to grazed plots, resulting in higher levels of humidity (Eskelinen et al., 2022). This heightened humidity can in turn drive changes in decomposition rates, soil properties, and biomass. Further research on vegetation‐soil dynamics is needed to understand the effect of duration of exclusion on biomass production.

Our results align with previous studies showing that the duration of grazing exclusion plays an important role in shaping vegetation dynamics. Thus, to manage grassland ecosystems in the long term, it is essential to understand vegetation recovery dynamics especially in relation to changes in soil properties following grazing exclusion.

### 4.5 Influence of climatic zone, evolutionary history of grazing, and grazing intensity on the effect of livestock exclusion on species richness, Shannon diversity, and aboveground biomass

We expected that grazing exclusion would decrease plant diversity only under moderate grazing intensities and/or in sites with drier conditions and with a shorter history of grazing. Although we have a small number of studies with moderate grazing (*n* = 2), we can confirm our expectation of decreased diversity under moderate grazing. This result is in line with the intermediate disturbance hypothesis (IDH) (Connell, 1978; Grime, 1973; Horn, 1975; Milchunas et al., 1988) posing that grasslands under moderate grazing can increase diversity by herbivores reducing competition for resources, allowing species coexistence until excessive grazing intensity becomes a disturbance. However, we did not find a link between climatic zone, evolutionary history of grazing or herbivore type on plant diversity, or aboveground biomass. This contrasts with previous studies suggesting differences in the effect of herbivory depending on aridity of the ecosystem (Borer et al., 2020; Gao & Carmel, 2020). We expected that subtropical grasslands in our study would be more prone to rapidly lose diversity under the effect of grazing due to higher aridity; however, the range of aridity was not large enough to detect these differences.

### 4.6 Limitation of the study

We conducted a thorough and inclusive review of studies in both Spanish and English related to our topic of interest. Despite an initial pool of 3121 studies in the first screening, only 13 met our specific criteria. The limited sample size resulting from this screening process prevents us from drawing robust conclusions about the impact of grazing on diversity and productivity in mountainous grasslands in South America. Throughout our search, we observed that Spanish literature search engines lack a comprehensive approach to identifying relevant studies. Despite our best efforts to include all published, peer‐reviewed studies in Spanish, we faced challenges due to the limitations of existing search tools.

Additionally, English‐language search engines have yet to incorporate numerous sources of literature in different languages. While Web of Science has integrated Scientific Electronic Library Online (SciELO) into its core collection for Spanish literature, there are other Spanish online libraries that could be considered. Despite these challenges, we trust that the number of studies included in our analysis accurately reflects the available research. However, it is essential to note that experimental studies explaining the mechanisms behind biodiversity patterns in the mountainous regions of South America are lacking.

This gap may be attributed to the inadequate infrastructure and funding necessary for conducting such experiments, particularly in the remote areas of South America where logistical challenges and costs are significant. Consequently, urgent collaborative efforts are needed between countries and researchers in the region to establish a network dedicated to advancing our understanding of the ecology of South American mountainous ecosystems. These ecosystems are crucial for the well‐being of millions of people, highlighting the importance of advancing research collaborations to address this knowledge gap.

## 5 CONCLUSIONS

Here we tested whether results from previous global meta‐analysis, mainly from temperate grasslands, apply to mountainous (sub)tropical grasslands. Our meta‐analysis confirms that grazing exclusion may reduce plant diversity and increase primary productivity. Thus, decreases in plant diversity and increases in primary productivity after livestock exclusion appear to be a universal pattern across grassland systems. However, our study suggests that the processes leading to changes in plant diversity in mountainous (sub)tropical grasslands differ from those observed in temperate grasslands. This may indicate that other non‐biomass processes not tested here might explain better the effects of livestock exclusion on plant biodiversity in these areas. We further established that longer duration of exclusion decreased Shannon diversity. This means that dominant palatable species might become more abundant with longer duration of exclusion. Overall, our findings have important conservation implications, because grazing exclusion by fence has become a common practice worldwide for managing grazed grasslands. In the case of mountainous grasslands of South America, conservation practitioners and local farmers might only achieve the recovery of above ground biomass in degraded grasslands after 5 years. However, they might see increases in the abundance of palatable species, which is favorable to maintain a healthy livestock production. Research is urgently needed to investigate further which processes, other than light limitation, are responsible for the vegetation dynamics in these grassland ecosystems to take appropriate conservation action.

## AUTHOR CONTRIBUTIONS

**Ana Patricia Sandoval‐Calderon:** Conceptualization (lead); data curation (lead); formal analysis (lead); funding acquisition (lead); investigation (lead); methodology (equal); project administration (lead); resources (lead); software (lead); supervision (equal); validation (equal); visualization (lead); writing – original draft (lead); writing – review and editing (lead). **Nerea Rubio Echazarra:** Data curation (equal); formal analysis (equal); writing – review and editing (supporting). **Marijke van Kuijk:** Supervision (supporting); writing – review and editing (supporting). **Pita A. Verweij:** Formal analysis (supporting); supervision (supporting); writing – review and editing (supporting). **Merel Soons:** Writing – original draft (supporting); writing – review and editing (supporting). **Yann Hautier:** Conceptualization (supporting); formal analysis (equal); investigation (supporting); methodology (supporting); software (supporting); supervision (supporting); writing – original draft (supporting); writing – review and editing (supporting).

## CONFLICT OF INTEREST STATEMENT

The authors declare that they have no known competing financial interests or personal relationships that could have appeared to influence the work reported in this paper.

## Supporting information


Appendix S1.

Data S1.

## Data Availability

The data that support the findings of this study are openly available in Zenedo at https://doi.org/10.5281/zenodo.10608874.
