# Supplementary material for: The effect of livestock grazing on plant diversity and productivity of mountainous grasslands in South America – A meta‐analysis
Source: Ecol Evol. 2024 Apr 15;14(4):e11076. doi: 10.1002/ece3.11076 (PMC11019300; doi:10.1002/ece3.11076)
Supplement: Supplementary file 1 — Appendix S1. [file ECE3-14-e11076-s001.docx]

**Supplementary material**

**AppendixS1**

**PICO elements for the definition of our search string**

| Subject | Intervention | Comparators | Outcomes |
| --- | --- | --- | --- |
| Natural and seminatural montane grasslands in tropical and subtropical regions of the Andes | Exclusion of domesticated and wild herbivores | Control plots (i.e., plots grazed by domesticated herbivores) | Species richness, Shannon diversity, and aboveground biomass |

**Appendix S2**

**Search string in English**

TS=((tropic* OR subtropic* OR alpine OR  montan* OR Argentin* OR Chile* OR Bolivia* OR Colombia* OR Peru OR Venezuela OR Equator*) AND ("grasslands" OR steppe OR gramin* OR bog OR peatbog OR rangeland OR meadow OR herbace* OR forb* OR heath OR wetland* OR paramo OR pampa* OR puna OR "moist puna" OR "xeric puna" OR tropic* OR subtropic*) AND (graz* OR herbivor* OR livestock OR husbandry OR landuse Or pastoralits* )  AND (enclousur* OR fenc* OR exclosur* OR "environment*"OR "rehabilitation" OR effect) AND (vegetat* OR flora OR plant*)AND (diversity OR richness OR species OR assemblag* OR  "species composition" ))

**Search string in Spanish**

TS=((tropic* OR trópic* OR neotropic* OR neotrópic* OR subtropic* OR subtrópic* OR andes OR “altos andes” OR altiplan* OR altoandin* OR alpino OR “tierras altas” OR “alta montaña” OR montan* OR montañ* OR páramo OR pampa OR subhumed* OR subhúmed* OR puna OR "puna húmeda" OR "puna norteña" OR "puna seca" OR "puna árida" OR "puna xerofítica" OR jalca OR “estepa patagónica” OR “meseta de alta montaña”)AND(herbivo* OR herví* OR pastoreo OR ganado OR “ganado doméstico” OR ganadería OR camélido OR intensi* OR mamífero OR ungulado OR “sistema pastoreado” OR “uso de la tierra” OR “cambio$ del uso de la tierra” OR “cambio$ en el uso de la tierra” OR “cambio$ de uso de la tierra” OR “intensificación del uso de la tierra” OR “intensificación en el uso de la tierra” OR “intensificación de uso de la tierra” OR recinto* OR cercado* OR valla* OR exclusión OR exclusiones OR manipula* OR “condiciones de pastoreo”)AND(“composición florística" OR “diversidad florística" OR “abundancia florística" OR “comunidad florística" OR “densidad florística" OR “riqueza florística” OR “patrón florístico” OR “patrones florísticos” OR “composición botánica” OR “composición del pastizal” OR “diversidad de plantas” OR “comunidad* de plantas” OR “especies de plantas” OR “conjunto de plantas” OR “tipos funcionales de plantas” OR vegeta* OR “diversidad vegeta*” OR “comunidad* vegeta*” OR “abundancia* vegeta*” OR “densidad vegeta*" OR “cobertura vegeta*" OR “biomasa veget*” OR productividad OR producción OR “composición de la biomasa” OR flora OR hierba* OR herbác* OR gramín* OR gramin* OR “diversidad de especie forrajeras” OR “especie* forrajera*”))

**Appendix S3. List of reference of the studies included in the meta-analysis**

1. Ayarde, H.R., & González, J.A. (2013). Recuperación de la vegetación natural por exclusión de pastoreo en una zona de montaña del noroeste de Argentina.
2. Barros, A., Pickering, C. M., & Renison, D. (2014). Short-term effects of pack animal grazing exclusion from Andean alpine meadows. Arctic, Antarctic, and Alpine Research, 46(2), 333-343.
3. Buttolph, L. P., & Coppock, D. L. (2004). Influence of deferred grazing on vegetation dynamics and livestock productivity in an Andean pastoral system. Journal of Applied Ecology, 41(4), 664-674.
4. Carilla, J., Aragón, R., & Gurvich, D.E. (2011). Fire and grazing differentially affect aerial biomass and species composition in Andean grasslands. *Acta Oecologica-international Journal of Ecology, 37*, 337-345.
5. Cingolani, A. M., Noy-Meir, I., & Díaz, S. (2005). Grazing effects on rangeland diversity: a synthesis of contemporary models. Ecological applications, 15(2), 757-773.
6. Danet, A., Anthelme, F., Gross, N., & Kéfi, S. (2018). Effects of indirect facilitation on functional diversity, dominance and niche differentiation in tropical alpine communities. Journal of Vegetation Science, 29(5), 835-846.
7. de Villalobos A.E., S.M. Zalba.2010. Continuous feral horse grazing and grazing exclusion in mountain pampean grasslands in Argentina. Acta Oecologica,Volume 36, Issue 5
8. Duchicela, S. A., Cuesta, F., PintoR…(2019). Indicators for assessing tropical alpine rehabilitation practices. Ecosphere, 10(2), e02595.
9. Marquez, S., Funes, G., Cabido, M., & Pucheta, E. (2002). Efectos del pastoreo sobre el banco de semillas germinable y la vegetación establecida en pastizales de montaña del centro de Argentina. *Revista chilena de historia natural*, *75*(2), 327-337.
10. Nai-Bregaglio, M., Pucheta, E., & Cabido, M. (2002). El efecto del pastoreo sobre la diversidad florística y estructural en pastizales de montaña del centro de Argentina. *Revista Chilena de Historia Natural*, *75*(3), 613-623.
11. Passera, Carlos B.; Allegretti, L. I.; Allegretti, L. I.; Borsetto, O. (1996) Respuesta de la vegetación excluida al pastoreo en una comunidad de Larrea cuneifolia del piedemonte mendocino Multequina, núm. 5, 1996, pp. 25-31
12. Pucheta, E., Cabido, M., Díaz, S., & Funes, G. (1998a). Floristic composition, biomass, and aboveground net plant production in grazed and protected sites in a mountain grassland of central Argentina. Acta Oecologica, 19(2), 97-105.
13. Pucheta, E., Díaz, S., & Cabido, M. (1992). The effect of grazing on the structure of a high plateau grassland in central Argentina. Coenoses, 145-152.

**Appendix S4. List of studies included in the meta-analysis, with study area, year of publication. G_Int= Grazing intensity. G_his= Grazing History. Hrb_type= Herbivore type. Grs_type= grassland system type**

| **Study** | **Year** | **Country** | | **Site** | **Language** | **Climatic Zone** | **Altitude** | **G_Int** | **G_his** | **Hrb_type** | **Grs_type** |
| --- | --- | --- | --- | --- | --- | --- | --- | --- | --- | --- | --- |
| Ayarde 1 | 2013 | Argentina | Agua Rica - Melcho | | Spanish | Subtropical | 3250 | Low | Short | Cattle | Tall grassland |
| Ayarde 2 | 2013 | Argentina | Agua Rica - Melcho | | Spanish | Subtropical | 3250 | Low | Short | Cattle | Tall grassland |
| Ayarde 3 | 2013 | Argentina | Agua Rica - Melcho | | Spanish | Subtropical | 3250 | Low | Short | Cattle | Tall grassland |
| Ayarde 4 | 2013 | Argentina | Agua Rica - Pozos | | Spanish | Subtropical | 2800 | Low | Short | Cattle | Tall grassland |
| Ayarde 5 | 2013 | Argentina | Agua Rica - Pozos | | Spanish | Subtropical | 2800 | Low | Short | Cattle | Tall grassland |
| Ayarde 6 | 2013 | Argentina | Agua Rica - Pozos | | Spanish | Subtropical | 2800 | Low | Short | Cattle | Tall grassland |
| Barros 1 | 2014 | Argentina | Aconcagua Provincial Park | | English | Subtropical | 3251 | High | Short | Cattle | Bofedal |
| Barros 2 | 2014 | Argentina | Aconcagua Provincial Park | | English | Subtropical | 3200 | High | Short | Cattle | Bofedal |
| Barros 3 | 2014 | Argentina | Aconcagua Provincial Park | | English | Subtropical | 3795 | High | Short | Cattle | Bofedal |
| Buttolph 1 | 2004 | Bolivia | Cosapa | | English | Tropical | 3970 | High | Long | Camelids | Bofedal |
| Buttolph 2 | 2004 | Bolivia | Cosapa | | English | Tropical | 3970 | High | Long | Camelids | Short grassland |
| Carilla 1 | 2011 | Argentina | Los Toldos valley | | English | Subtropical | 1600 | Low | Short | Cattle | Tall grassland |
| Carilla 2 | 2011 | Argentina | Los Toldos valley | | English | Subtropical | 1600 | Low | Short | Cattle | Tall grassland |
| Cingolani | 2003 | Argentina | Pampa de Achala | | English | Subtropical | 1800-2300 | Low | Short | Cattle | Tall grassland |
| Danet | 2017 | Bolivia | Palcoco valley | | English | Tropical | 4000-5000 | Low | Long | Camelids | Bofedal |
| de Villalobos | 2010 | Argentina | Ventania Moutain Range | | English | Subtropical | 1200 | High | Short | Cattle | Tall grassland |
| Duchicela 1 | 2019 | Perú | Huancavelica | | English | Tropical | 4564 | High | Long | Camelids | Bofedal |
| Duchicela 2 | 2019 | Perú | Huancavelica | | English | Tropical | 4480 | High | Long | Camelids | Short grassland |
| Marquez | 2002 | Argentina | Pampa de Achala | | Spanish | Subtropical | >2000 | Moderate | Short | Cattle | Tall grassland |
| Nai-Bregaglio | 2002 | Argentina | Pampa de Achala | | Spanish | Subtropical | 2200 | Moderate | Short | Cattle | Tall grassland |
| Passera 1 | 1996 | Argentina | Puesto "La Pichana" | | Spanish | Subtropical | 1075 | High | Short | Cattle | Tall grassland |
| Passera. 2 | 1996 | Argentina | Puesto "La Pichana" | | Spanish | Subtropical | 1075 | High | Short | Cattle | Tall grassland |
| Passera 3 | 1996 | Argentina | Puesto "La Pichana" | | Spanish | Subtropical | 1075 | High | Short | Cattle | Tall grassland |
| Pucheta | 1992 | Argentina | Pampa de Achala | | English | Subtropical | 2250 | High | Short | Cattle | Tall grassland |
| Pucheta (b) 1 | 1998 | Argentina | Pampa de Achala | | English | Subtropical | 2150 | High | Short | Cattle | Tall grassland |
| Pucheta (b) 2 | 1998 | Argentina | Pampa de Achala | | English | Subtropical | 2150 | High | Short | Cattle | Tall grassland |
| Pucheta(b) 3 | 1998 | Argentina | Pampa de Achala | | English | Subtropical | 2150 | High | Short | Cattle | Tall grassland |
